# A hinged external fixator for complex elbow dislocations: A multicenter prospective cohort study

**DOI:** 10.1186/1471-2474-12-130

**Published:** 2011-06-09

**Authors:** Niels WL Schep, Jeroen De Haan, Gijs IT Iordens, Wim E Tuinebreijer, Maarten WGA Bronkhorst, Mark R De Vries, J Carel Goslings, S John Ham, Steven Rhemrev, Gert R Roukema, Inger B Schipper, Jan Bernard Sintenie, Hub GWM Van der Meulen, Tom PH Van Thiel, Arie B Van Vugt, Egbert JMM Verleisdonk, Jos PAM Vroemen, Philippe Wittich, Peter Patka, Esther MM Van Lieshout, Dennis Den Hartog

**Affiliations:** 1 Department of Surgery-Traumatology, Erasmus MC, University Medical Center Rotterdam, P.O. Box 2040, 3000 CA Rotterdam, The Netherlands; 2 Department of Surgery-Traumatology, Westfriesgasthuis, P.O. Box 600, 1620 AR Hoorn, The Netherlands; 3 Department of Surgery-Traumatology, Bronovo Hospital, P.O. Box 96900, 2509 JH The Hague, The Netherlands; 4 Department of Surgery-Traumatology, Reinier de Graaf Gasthuis, P.O. Box 5011, 2600 GA Delft, The Netherlands; 5 Trauma Unit Department of Surgery, Academic Medical Center, P.O. Box 22660, 1100 DD Amsterdam, The Netherlands; 6 Department of Orthopaedic Surgery, Onze Lieve Vrouwe Gasthuis, P.O. Box 95500, 1090 HM Amsterdam, The Netherlands; 7 Department of Surgery-Traumatology, Medical Center Haaglanden, P.O. Box 432, 2501 CK 's-Gravenhage, The Netherlands; 8 Department of Surgery-Traumatology, Maasstad Hospital, P.O. Box 9100, 3007 AC Rotterdam, The Netherlands; 9 Department of Surgery-Traumatology, Leiden University Medical Center, P.O. Box 9600, 2300 RC Leiden, The Netherlands; 10 Department of Surgery-Traumatology, Elkerliek Hospital, P.O. Box 98, 5700 AB Helmond, The Netherlands; 11 Department of Surgery-Traumatology, Haga Hospital, P.O. Box 40551, 2504 LN's- Gravenhage, The Netherlands; 12 Department of Surgery-Traumatology, Hospital Queen Beatrix, P.O. Box 9005, 7100 GG Winterswijk, The Netherlands; 13 Department of Surgery-Traumatology, Medical Spectrum Twente, P.O. Box 50.000, 7500 KA Enschede, The Netherlands; 14 Department of Surgery-Traumatology, Diakonessenhuis, P.O. Box 80250, 3508 TG Utrecht, The Netherlands; 15 Department of Surgery-Traumatology, Amphia Hospital, P.O. Box 90158, 4800 RK Breda, The Netherlands; 16 Department of Surgery-Traumatology, Sint Antonius Hospital, P.O. Box 2500, 3430 EM Nieuwegein, The Netherlands

## Abstract

**Background:**

Elbow dislocations can be classified as simple or complex. Simple dislocations are characterized by the absence of fractures, while complex dislocations are associated with fractures of the radial head, olecranon, or coronoid process. The majority of patients with these complex dislocations are treated with open reduction and internal fixation (ORIF), or arthroplasty in case of a non-reconstructable radial head fracture. If the elbow joint remains unstable after fracture fixation, a hinged elbow fixator can be applied. The fixator provides stability to the elbow joint, and allows for early mobilization. The latter may be important for preventing stiffness of the joint. The aim of this study is to determine the effect of early mobilization with a hinged external elbow fixator on clinical outcome in patients with complex elbow dislocations with residual instability following fracture fixation.

**Methods/Design:**

The design of the study will be a multicenter prospective cohort study of 30 patients who have sustained a complex elbow dislocation and are treated with a hinged elbow fixator following fracture fixation because of residual instability. Early active motion exercises within the limits of pain will be started immediately after surgery under supervision of a physical therapist. Outcome will be evaluated at regular intervals over the subsequent 12 months. The primary outcome is the *Quick *Disabilities of the Arm, Shoulder, and Hand score. The secondary outcome measures are the Mayo Elbow Performance Index, Oxford Elbow Score, pain level at both sides, range of motion of the elbow joint at both sides, radiographic healing of the fractures and formation of periarticular ossifications, rate of secondary interventions and complications, and health-related quality of life (Short-Form 36).

**Discussion:**

The outcome of this study will yield quantitative data on the functional outcome in patients with a complex elbow dislocation and who are treated with ORIF and additional stabilization with a hinged elbow fixator.

**Trial Registration:**

The trial is registered at the Netherlands Trial Register (NTR1996).

## Background

The elbow joint is the second most commonly dislocated joint in adults. The annual incidence of elbow dislocations in children and adults is 6.1 per 100,000 [1]. Elbow dislocations are classified as being simple or complex [2]. Simple dislocations are dislocations without fractures. Complex dislocations are associated with fractures of the radial head, olecranon, or coronoid process. In patients with an elbow dislocation the incidence of radial head fractures is 36%, whereas coronoid process fractures occur in 13%, and olecranon fractures in four percent of patients [1].

The radial head and coronoid process are considered to be important bony stabilizers of the elbow. The fundamental goal in the management of complex elbow dislocations is the restoration of the osseous-articular restraints. Therefore, the majority of these complex dislocations is treated with open reduction and internal fixation (ORIF) [3] or primary arthroplasty in case of a non-reconstructable radial head fracture.

Assessment of stability of the joint following ORIF of a complex elbow dislocation is essential. Signs of instability are redislocation, a positive pivot shift test and positive valgus and varus stress testing. At present instability following ORIF or arthroplasty is usually treated with primary ligament repair and/or a period of plaster immobilization.

A period of plaster immobilization may result in a limited range of motion and a stiff elbow with subsequent disability. A hinged external elbow fixator, on the other hand, may provide enough stability to start early mobilization after ORIF or arthroplasty and may prevent residual instability and stiffness [4,5]. No randomized controlled trials comparing hinged external fixation and plaster immobilization are available. This may be due to the low incidence of patients with a complex elbow dislocation with remaining instability after ORIF or arthroplasty. Until now only small observational studies of patients with complex elbow dislocations have been published [2,3,5-12]. These studies showed promising functional results following treatment with a hinged elbow fixator [11,12].

The primary objective of this prospective cohort study is to study the functional outcome, pain, and health-related quality of life in patients who sustained a complex elbow dislocation and were treated with ORIF and/or arthroplasty of the radial head and a hinged external fixator due to residual instability. Our hypothesis is that early mobilization will prevent stiffness and will result in a satisfactory functional outcome at one year.

## Methods/Design

### Study design

Multi-center cohort study in all consecutive patients who sustained a complex elbow dislocation and were treated with a hinged external fixator for residual instability after ORIF and/or arthroplasty of the radial head. Sixteen centers in the Netherlands will participate. The study started August 28, 2009.

### Recruitment and consent

The decision to apply the hinged fixator for residual instability following fracture fixation will be left to the discretion of the surgeon. If a fixator is applied, patients will receive information and a consent form from the attending physician, the clinical investigator or a research assistant postoperatively. Patients meeting all inclusion criteria and none of the exclusion criteria will be included before discharge or at the time of their first outpatient visit (two weeks after surgery), which will give them on average one week to consider their participation.

### Study population

Patients meeting the following inclusion criteria are eligible for enrolment:

1. Men or women aged 18 years and older (with no upper age limit)

2. Patient with a complex elbow dislocation (i.e., dislocation of the elbow joint, combined with at least a fracture of the radial head, coronoid process, or olecranon)

3. Patient was treated with a hinged external fixator after ORIF and/or arthroplasty of the radial head due to persistent instability

4. Provision of informed consent by patient

Since there is currently no consensus regarding the most valid and reliable test for assessing elbow joint instability, this will be left to the discretion of the surgeon performing the operation. This reflects common practice, and will increase translatability of the outcome of our study. In order to warrant performance of stability tests across participating sites, a detailed description of stability tests (*i.e*., varus stability, valgus stability, and pivot shift test for posterolateral rotatory stability) is included in the protocol.

If any of the following criteria applies, patients will be excluded:

1. Patients with a concomitant distal humeral fracture

2. Patients with additional substantial traumatic injuries of the affected upper limb

3. Patients who underwent repair of the collateral ligaments

4. Patients with an impaired elbow function (i.e., stiff or painful elbow or neurological disorder of the upper limb) prior to the injury

5. Retained hardware around the affected elbow

6. Likely problems, in the judgment of the investigators, with maintaining follow-up (e.g., patients with no fixed address will be excluded)

7. Insufficient comprehension of the Dutch language to understand the rehabilitation program and other treatment information in the judgment of the attending physician

Exclusion of a patient because of enrolment in another ongoing drug or surgical intervention trial will be left to the discretion of the attending surgeon on a case-by-case basis.

### Intervention

The external fixator used is the Orthofix^® ^Elbow Fixator (Orthofix Verona, Italy). The surgical approach to the fracture site is left to the surgeon's discretion. Following ORIF of the fractures and/or arthroplasty of the radial head, the center of rotation of the elbow is identified. A two mm K-wire is inserted into the center point of the capitellum humeri which is identified on an exact lateral fluoroscopic image. Next, the external fixator is mounted, first fixating the proximal humeral clamp and subsequently the distal ulnar clamp. Exact reduction of the elbow joint is evaluated with image intensifier in lateral and anteroposterior direction during flexion and extension. The surgical technique is described in more details elsewhere [13]. After surgery, patients are allowed to use a sling for two days to one week. Pin-site care will be performed daily by the patient following instruction given by the treating physician. After surgery patients will receive indomethacin 2dd 50 mg for six weeks (in combination with acid blocking medication) in order to prevent heterotopic ossification of the elbow, unless NSAIDs are contraindicated [14]. The external fixator will be removed six weeks after surgery. Extension, flexion and pro- and supination active and passive exercises are started immediately after surgery if tolerated under supervision of a professional physical therapist, who they can freely select.

### Outcome measures

The primary outcome measure is the *Quick-*DASH (Disabilities of the Arm, Shoulder and Hand) score, which reflects both function and pain after one year [15]. The DASH Outcome Measure is a validated 30-item, self-reported questionnaire designed to help describe the disability experienced by people with upper-limb disorders and also to monitor changes in symptoms and function over time [15,16].

The *Quick-*DASH is a shortened version of the DASH Outcome Measure. Instead of 30 items, the Quick-DASH uses 11 items (scored 1-5) to measure physical function and symptoms in people with any or multiple musculoskeletal disorders of the upper limb. The right and left elbow will be assessed separately. At least 10 of the 11 items must be completed for a score to be calculated. The scores will be transformed to a 0-100 scale for easy comparison. A higher score indicates greater disability. The test-retest reliability of the *Quick*-DASH was 0.90 [17].

Like the DASH, the *Quick-*DASH also has two optional modules intended to measure symptoms and function in athletes, performing artists and other workers whose jobs require a high degree of physical performance. These optional models are scored separately; each contains four items, scored 1-5. All items must be completed for a score to be calculated.

The secondary outcome measures are:

• Functional outcome (Mayo Elbow Performance Index and Oxford Elbow Score)

• Pain level at both sides (VAS)

• Range of Motion of the elbow joint at both sides

• Radiographic healing of the fractures

• Rate of secondary interventions

• Rate of complications

• Health-related quality of life (SF-36)

The Mayo Elbow Performance Index (MEPI) is one of the most commonly used physician-based elbow rating systems. This index consists of five parts: pain (with a maximum score of 45 points), ulnohumeral motion (20 points), stability (ten points), the ability to perform five functional tasks (5 × 5 points) and the patient response. If the total score is between 90 and 100 points, it is considered excellent; between 75 and 89 points, good; between 60 and 74 points, fair; and less than 60 points, poor [18].

The Oxford Elbow Score is a 12-item questionnaire. It is comprised of three one-dimensional domains: elbow function, pain and social-psychological, with each domain comprising of four items with good measurement properties [19]. This is a validated questionnaire in the UK and was translated to Dutch by the proper translation procedure, which uses the technique of translation and back-translation [20-22]. Permission for translation and the use of the Oxford Elbow Score for this study was obtained from Oxford and Isis Outcomes, part of Isis Innovation Limited (website: http://www.isis-innovation.com/)

Pain level will be determined using a 10-point Visual Analog Scale (VAS), in which zero implies no pain and ten implies the worst possible pain.

Range of motion (ROM) will be determined by measure flexion/extension and pro-/supination on both sides using a goniometer.

Radiographic healing will be determined using X-rays. Fractures are considered healed if one of the following three criteria is met: (a) Bridging of fracture by callus/bone trabeculae or osseous bone; (b) Obliteration of fracture line/cortical continuity; (c) Bridging of fracture at three cortices.

Secondary intervention within one year of initial treatment to promote fracture healing, relieve pain, treat infection, or improve function will be recorded. This includes incision and drainage for surgical site infection or deep infection, repositioning or removal of the fixator, reosteosynthesis, implant removal, or ligament repair.

Complications within one year of initial treatment will be recorded. These include heterotopic ossification, infections, bleeding, venous thrombosis, and neurological deficits)

The Short-Form 36 (SF-36) is a validated multi-purpose, short-form health survey with 36 questions that represent eight health domains that are combined into a physical and a mental component scale [23]. The Physical Component Scale (PCS) combines the health domains of physical functioning (PF; ten items), role limitations due to physical health (RP; four items), bodily pain (BP; two items), and general health perceptions (GH; five items). The Mental Component Scale (MCS) combines the health domains of vitality, energy, or fatigue (VT; four items), social functioning (SF; two items), role limitations due to emotional problems (RE; three items), and general mental health (MH; five items). Scores ranging from zero to 100 points are derived for each domain, with lower scores indicating poorer function. These scores will be converted to a norm-based score and compared with the norms for the general population of the United States (1998), in which each scale was scored to have the same average (50 points) and the same standard deviation (ten points).

In addition to the outcome variables mentioned above, the following data will be collected:

1. Intrinsic variables (baseline data): age, gender, American Society of Anesthesiologists' ASA classification, tobacco consumption, alcohol consumption, comorbidity, dominant side, medication use, *Quick-*DASH score prior to the injury, pain level at both sides prior to the injury (VAS), and SF-36 score prior to the injury.

2. Injury related variables: affected side, mechanism of injury, and postoperative assessment of varus, valgus and posterolateral rotatory instability, fracture location (*i.e*., radial head, coronoid process, olecranon), fracture classification of the coronoid process according to Regan & Morrey [24], and fracture classification of the radial head according to Mason & Johnston [25].

3. Intervention-related variables: surgical delay (*i.e*., time between fracture and surgery), time between injury and start of physical therapy, and number of physical therapy sessions

### Study procedures [Table 1]

**Table 1:** Schedule of events

	Screening	Enrolment	Baseline	< 48 h post-surgery	2 weeks (7-28 d)	6 weeks (4-8 we)	3 months (11-15 we)	6 months (5-7 mo)	12 months (12-14 mo)
**Screening**	**X**								

**X-ray**	**X**			**X**	**X**	**X**	**X**	**X**	**X**

**Informed Consent**		**X**							

**Baseline data**			**X**						

***Quick-*DASH**			**X**			**X**	**X**	**X**	**X**

**Pain (VAS)**			**X**		**X**	**X**	**X**	**X**	**X**

**SF-36**			**X**			**X**	**X**	**X**	**X**

**Clinical follow-up**					**X**	**X**	**X**	**X**	**X**

**Revision surgery**					**X**	**X**	**X**	**X**	**X**

**Complications**					**X**	**X**	**X**	**X**	**X**

**ROM**					**X**	**X**	**X**	**X**	**X**

**MEPI**						**X**	**X**	**X**	**X**

**Oxford Elbow Score**						**X**	**X**	**X**	**X**

**Early withdrawal**				*	*	*	*	*	*

*, only if applicable

Clinical assessments will take place at the time of admission to the hospital (baseline), two weeks (7-28 days window), six weeks (4-8 weeks window), three months (11-15 weeks window), six months (5-7 months window), and 12 months (12-14 months window) after surgery. At each follow-up moment, the research coordinator or research assistant will ascertain patient status (*i.e*., secondary interventions, adverse events/complications), and will verify information within medical records. At the last visit, the surgeon will document any surgery that may be planned for the patient.

Anteroposterior and lateral X-rays of the elbow will be made at the time of presentation to the hospital (baseline), within 48 hours post surgery, and at all follow-up visits listed above. These X-rays will be used to determine the time to radiographic healing and amount and location of heterotopic ossification.

At baseline, patients will be asked to complete the *Quick-*DASH, VAS, and SF-36 questionnaires. This relates to the situation prior to the injury, so in order to minimize recall bias as much as possible, the questionnaires will be completed as soon after surgery as possible. At the two weeks follow-up visit and each visit thereafter, the range of motion of the elbow joint will be measured by a doctor or research assistant using a goniometer. At these follow-up visits, the patients will complete a questionnaire relating to pain (VAS). The MEPI index will be determined from six weeks onwards. At the six week follow-up visit and each visit thereafter patients will be asked to complete the *Quick-*DASH, Oxford Elbow Score, and SF-36 questionnaires.

### Sample size calculation

Calculation of the required sample size for this study is not constructive. This study is a case series based on the assumption that for introducing and acquiring experience in a new operative technique a sample size of 30 patients is required [26,27].

### Statistical analysis

Data will be analyzed using the PASW Statistics version 18.0.1 or higher (SPSS, Chicago, Illinois, USA). Normality of continuous data will be checked by inspecting the frequency distributions (histograms) and normal Q-Q plots. Data will be reported in compliance with the CONSORT (CONsolidation of Standards of Reporting Trials) guidelines [28,29]. In the unlikely event that a fixator will be removed within six weeks, patients will be followed and analyzed on an intention to treat basis.

Descriptive analysis will be performed in order to report baseline characteristics (*i.e*., intrinsic, injury-related and fracture-related variables) and outcome measures. For continuous variables (*e.g*., age, *Quick-*DASH score, MEPI, VAS, and SF-36 score) mean ± SD (if normally distributed) or medians and percentiles (if not normally distributed) will be calculated. For categorical variables (*e.g*., gender, ASA grade, alcohol and tobacco consumption, dominant and affected side) frequencies will be calculated.

Multiple linear regression analysis will be performed in order to model the relation between different covariates and the *Quick-*DASH score. Intrinsic and fracture-related variables will be added as covariate. Similar models will be made to model the relation between covariates and the other outcome measures. A p-value < 0.05 will be taken as the threshold of statistical significance.

### Ethical considerations

The study will be conducted according to the principles of the Declaration of Helsinki (59th World Medical Association General Assembly, Seoul, October 2008) and in accordance with the Medical Research Involving Human Subjects Act (WMO).

The Medical Ethics Committee Erasmus MC (Rotterdam, The Netherlands) acts as central ethics committee for this trial (reference number MEC-2009-240; NL28503.078.09). Approval has been obtained from the local Medical Ethics Committees in all participating centers. Obtaining medical ethics approval has coordinated and organized by a central research coordinator (EMMVL), who is part of the key investigator team and employed by the initiating site Erasmus MC. She prepared all documents for the participating sites and answered questions of the local ethics committees if there were any. This was always following review and approval of the site principal investigator. All participating surgeons have had GCP training previously or were trained at the initiation visit in order to meet legal requirements.

An information letter notifying the patients' participation will be sent to their general practitioners, unless a patient does not agree with this.

The Medical Ethics Committee Erasmus MC has given dispensation from the statutory obligation to provide insurance for subjects participating in medical research (article 7, subsection 6 of the WMO and Medical Research (Human Subjects) Compulsory Insurance Decree of 23 June 2003). The reason for this dispensation is that participation in this study is without risks.

## Discussion

The outcome of this study will yield quantitative data on the functional outcome patients with a complex elbow dislocation and who are treated with ORIF and additional stabilization with a hinged elbow fixator. Early functional treatment may lead to a better ROM and prevent elbow stiffness. Furthermore, the data as collected during this study may be used for designing future (randomized) clinical trials. Inclusion of patients has been started August 28, 2009 and the expectation is to include 2-3 patients per month. With a follow-up of one year the presentation of data will be expected at the end of 2012.

## List of abbreviations used

ASA: American Society of Anesthesiologists; BP: Bodily Pain; CONSORT: CONsolidated Standards of Reporting Trial; DASH: Disabilities of the Arm, Shoulder and Hand score; GH: General Health perception; MCS: Mental Component Scale; MEPI: Mayo Elbow Performance Index; MH: general Mental Health; NTR: Netherlands Trial Registry (in Dutch: Nederlands Trial Register); ORIF: Open Reduction and Internal Fixation; PCS: Physical Component Scale; PF: Physical Functioning; RE: Role limitations due to Emotional problems; ROM: Range Of Motion; RP: role limitations due to physical health; SF: Social Functioning; SF-36: Short Form 36; SPSS: Statistical Package for the Social Sciences; VAS: Visual Analog Scale; VT: vitality, energy, or fatigue.; WMO: Medical Research Involving Human Subjects Act (in Dutch: Wet Medisch-wetenschappelijk Onderzoek met mensen).

## Competing interests

The authors declare that they have no competing interests.

## Authors' contributions

NWLS, JDH, WET, EMMVL, and DDH developed the trial and drafter the manuscript. DDH will act as trial principal investigator. WET, EMMVL and NWLS will perform statistical analysis of the trial data. NWLS, JDH, GITI, MWGAB, MRDV, JCG, SJH, SR, GRR, IBS, JBS, HGWMVDM, TPHVT, ABVV, EJMMV, JPAMV, PW, PP, and DDH will participate in patient inclusion and assessment. All authors have read and approved the final manuscript.

## Specified notice

Oxford Elbow Score^© ^Isis Innovation Limited, 2008. All rights reserved. The authors, being Professor Ray Fitzpatrick and Dr Jill Dawson, have asserted their moral rights.

## Pre-publication history

The pre-publication history for this paper can be accessed here:

http://www.biomedcentral.com/1471-2474/12/130/prepub
